# Fitness and ERP Indices of Cognitive Control Mode during Task Preparation in Preadolescent Children

**DOI:** 10.3389/fnhum.2016.00441

**Published:** 2016-08-30

**Authors:** Keita Kamijo, Hiroaki Masaki

**Affiliations:** Faculty of Sport Sciences, Waseda UniversityTokorozawa, Japan

**Keywords:** childhood fitness, cognitive control mode, proactive control, reactive control, AX-continuous performance task (AX-CPT), contingent negative variation (CNV), task preparation process

## Abstract

A growing number of studies conducted over the past decade have demonstrated that greater aerobic fitness is associated with superior cognitive control in preadolescent children. Several studies have suggested that the relationship between fitness and cognitive control may be attributed to differential reliance on proactive vs. reactive cognitive control modes. However, this contention has remained speculative, and further studies are needed to better elucidate this relationship. We designed the present study to test the hypothesis that use of cognitive control modes would differ as a function of childhood fitness. We compared performance of lower-fit and higher-fit children on a modified AX-continuous performance task, commonly used to examine shifts in the use of proactive and reactive control, along with cue-P3 and contingent negative variation (CNV) of event-related brain potentials (ERPs). Results indicated that higher-fit children exhibited greater response accuracy for BX (non-target cue – target probe) relative to AY (target cue – non-target probe) trials, whereas lower-fit children had comparable response accuracies for AY and BX trials. Because enhanced BX performance and impaired AY performance may be attributed to the proactive use of context information, these results suggest that greater childhood fitness is associated with more effective utilization of proactive control. Higher-fit children also exhibited larger cue-P3 amplitude and smaller CNV amplitude for BX relative to AY trials, with no such effect of trial type in lower-fit children. These ERP results suggest that greater fitness is associated with more effective utilization of cue information and response preparation more appropriate to trial type, supporting the behavioral findings. The present study provides novel insights into the relationship between fitness and cognition from the perspective of cognitive control mode during task preparation.

## Introduction

A growing number of cross-sectional studies have demonstrated that greater childhood fitness is associated with superior cognitive functioning, and this positive association is disproportionately greater for higher-order cognitive functions (i.e., cognitive control), such as inhibition, working memory, and cognitive flexibility (Hillman et al., 2009; Chaddock et al., 2010; Pontifex et al., 2011; Voss et al., 2011; Scudder et al., 2014). These cross-sectional results are supported by longitudinal, randomized controlled intervention studies indicating that a 9-month physical activity intervention improves cognitive control as well as aerobic fitness in preadolescent children (Kamijo et al., 2011; Hillman et al., 2014). These findings imply that childhood fitness is associated with prefrontal cortex (PFC) development, including the cognitive control network, as it is well established that the PFC plays a critical role in the effective regulation of cognitive control (Miller and Cohen, 2001). Although the underlying mechanisms for the beneficial effects of physical activity and fitness on cognitive control remained unclear, animal studies suggest that aerobic exercise-induced neurochemical changes – for example, in brain-derived neurotrophin factor and insulin-like growth factor 1 (Neeper et al., 1995; Carro et al., 2001) – increase neurogenesis and improve learning performance (van Praag et al., 1999). If the neurochemical changes with aerobic exercise seen in animal studies occur in the human brain and enhance PFC development, then such changes may be the reason, we find parallel improvement in cognitive control and aerobic fitness in children.

Several studies have suggested that the relationship between childhood fitness and cognitive control can be attributed to differential reliance on proactive vs. reactive modes of cognitive control (Kamijo et al., 2011, 2016; Pontifex et al., 2011; Voss et al., 2011; Berchicci et al., 2015), a pattern that is linked to PFC activity (Braver et al., 2007; Braver, 2012). However, this contention has remained speculative, and further studies are needed to better elucidate the relationship between childhood fitness and cognitive control. We designed the present study to test the hypothesis that the utilization of cognitive control mode varies as a function of childhood fitness.

According to the dual mechanisms of control framework (Braver et al., 2007; Braver, 2012), proactive control is reflected in sustained lateral PFC activation to actively maintain goal-relevant information, whereas reactive control is reflected in transient activation of the lateral PFC as well as a wider brain network to reactivate goal-related information on an as-needed basis. For example, during the classic Stroop color-naming task, participants who engage in more proactive control exhibit greater sustained PFC activation, which reflects active maintenance of task goals (i.e., to attend to color and ignore word information). By contrast, participants engaging in more reactive control exhibit a decrease in sustained PFC activation and an increase in transient activity in the PFC and other brain regions, such as the anterior cingulate cortex, that reflect transient stimulus-driven reactivation of task goals. The sustained, active maintenance of task goals (i.e., proactive control) reduces conflict, resulting in a smaller interference effect (De Pisapia and Braver, 2006; cf. Figure 1 of Braver, 2012). That is, “proactive control relies upon the anticipation and prevention of interference before it occurs, whereas reactive control relies upon the detection and resolution of interference after its onset” (Braver, 2012, p. 106).

We compared performance of lower-fit and higher-fit children on a modified AX-continuous performance task (AX-CPT), commonly used to examine shifts in the use of proactive and reactive control (Braver, 2012). The AX-CPT requires participants to respond (e.g., by means of a button press with the index finger) to a certain cue-probe pair (i.e., target cue – target probe; AX trials), and to withhold their response or make an alternate response (e.g., a button press with the middle finger) to other cue-probe pairs. These alternate cue-probe pairs, or non-target trials^1^, comprise three trial types: AY (A – non-X, target cue – non-target probe), BX (non-A – X, non-target cue – target probe), and BY (non-A – non-X, non-target cue – non-target probe) trials. Performance on the AY and BX trials can be affected by cognitive control mode (Paxton et al., 2006; Edwards et al., 2010; Braver, 2012). When the majority of stimulus presentations are AX trials (e.g., 64% of the total number of trials), participants are biased to respond to the target cue (i.e., AY trials) or the target probe (BX trials), even on non-target trials where such a response is contraindicated. On BX trials, the proactive use of context information should improve performance by inhibiting a target response bias. That is, if participants can utilize contextual information provided by the B cue, they can inhibit the prepotent target response and make appropriate responses on BX trials. By contrast, on AY trials, the proactive use of context information should create an expectancy to make a target response following an A cue, which in turn results in impaired performance. Thus, individuals who engage in more cue-driven proactive control should show superior performance on BX relative to AY trials. To sum up, the use of these alternative control modes can be assessed by comparing AY and BX performance (Paxton et al., 2006; Edwards et al., 2010).

The AX-CPT allows for measurement of task preparation processes using the cue-P3 and contingent negative variation (CNV; Jonkman, 2006; Hammerer et al., 2010) of event-related brain potentials (ERPs). The P3 is a positive ERP component occurring approximately 300–800 ms after stimulus onset and has a parietal distribution. P3 amplitude is believed to index the amount of attentional resources deployed during stimulus engagement (Polich, 2007). During the AX-CPT, cue-elicited P3 is associated with cue utilization. Specifically, target cues elicit larger P3 amplitude relative to non-target cues, indicating greater allocation of attentional resources (Jonkman, 2006; Hammerer et al., 2010). The CNV is a negative slow potential that develops during the interval between warning (i.e., cue) and imperative (i.e., probe) stimuli. It has been suggested that frontal CNV reflects cognitive preparation processes, whereas central CNV is associated with response preparation processes (Leynes et al., 1998; Lorist et al., 2000; Falkenstein et al., 2003; Wild-Wall et al., 2007; Kamijo et al., 2010, 2011). A number of studies have indicated that larger CNV amplitude is associated with better task performance (e.g., Hohnsbein et al., 1998; Smith et al., 2006). Taken together, this evidence suggests that greater utilization of proactive control should be reflected in larger cue-P3 amplitude (i.e., greater utilization of cue information) and larger CNV amplitude (i.e., more effective task preparation; Pincham et al., 2012), and this effect on CNV amplitude should be more pronounced over the frontal regions, reflecting cognitive preparation.

The present study was designed to examine the relationship between childhood fitness and cognitive control mode by measuring the cue-P3 and CNV during the AX-CPT. We employed a cross-sectional design, comparing task performance measures and ERP components across lower-fit and higher-fit children. Participants’ fitness was assessed using the Progressive Aerobic Cardiovascular Endurance Run (PACER; also referred to as the 20-m shuttle run test). The PACER is a widely used field test of aerobic capacity, which has demonstrated high test-retest reliability and validity against directly measured maximal oxygen uptake, which is the gold standard measure of aerobic fitness (Leger et al., 1988; Olds et al., 2006).

Developmental studies have indicated that proactive control develops from childhood to young adulthood but is delayed relative to reactive control (Chatham et al., 2009; Andrews-Hanna et al., 2011). Thus, we hypothesized that greater childhood fitness would be associated with more effective utilization of proactive control. As mentioned above, proactive use of context information is reflected in enhanced BX performance but impaired AY performance. Accumulating evidence further suggests that greater aerobic fitness is associated with superior cognitive performance (Hillman et al., 2009; Chaddock et al., 2010; Pontifex et al., 2011; Voss et al., 2011; Scudder et al., 2014). Accordingly, we predicted that higher-fit children would exhibit superior task performance for BX trials relative to lower-fit children, whereas such differences would be reduced for AY trials. We further predicted that higher-fit children would exhibit superior task performance on BX relative to AY trials, whereas this difference would be less pronounced for lower-fit children. With regard to ERP components, we predicted that higher-fit children would exhibit larger cue-P3 and CNV amplitudes relative to lower-fit children, and the difference in CNV amplitude would be more pronounced at frontal rather than central electrode sites. Finally, we also assessed probe-elicited N2 and P3, which are believed to reflect conflict monitoring and response suppression, respectively (Bruin et al., 2001; Nieuwenhuis et al., 2003; Jonkman, 2006; Hammerer et al., 2010). If the expected differences in task performance were due to greater utilization of proactive control (i.e., anticipation and prevention of interference before it occurs) by higher-fit children, we predicted that no group differences would be observed for the probe-elicited ERPs.

## Materials and Methods

### Participants

Forty-eight preadolescent children completed the AX-CPT. The participants also performed a modified flanker task. Because, we used the flanker task to investigate a different cognitive process (i.e., action monitoring), these data were reported elsewhere (Kamijo et al., 2016). Data from three obese children, as defined by the national cutoff point (Japanese Ministry of Education, Culture, Sports, Science, and Technology, 1981–2002), were excluded from the analyses, since it has been found that childhood obesity is negatively associated with cognitive control (Kamijo et al., 2012, 2014). In addition, data from seven participants were discarded due to excessive noise in the electroencephalographic (EEG) signal. Thus, analyses were conducted for 38 participants, and a median split was used to divide the participants into lower-fit and higher-fit groups on the basis of PACER percentile scores within each sex. **Table 1** lists the participants’ demographic and fitness information. The demographic measures, except for the fitness measures, did not differ between groups, *t*s (36) ≤ 1.1, *p*s ≥ 0.27. Prior to testing, legal guardians reported that their children were free of neurological diseases or physical disabilities and had normal or corrected-to-normal vision. None of the children received special education services related to cognitive or attentional disorders. All participants and their legal guardians provided written informed consent in accordance with the Ethics Committee on Human Research of Waseda University.

**Table 1:** Mean (*SD*) values for participant demographics and fitness data.

Measure	Lower-fit	Higher-fit
No. of participants	19 (9 girls)	19 (9 girls)
Age (years)	10.7 (1.2)	10.6 (0.8)
PACER (no. of laps)	37.3 (13.9)	67.5 (15.8)
PACER (percentile)	27.7 (18.3)	80.1 (15.0)
Body mass index (kg/m^2^)	17.0 (2.1)	16.4 (1.5)
Maternal education	2.9 (0.8)	2.8 (0.9)
ADHD	8.6 (6.9)	7.5 (6.0)

*Maternal education was assessed using a five-point scale ranging from, 1 indicating that they did not complete high-school, to 5, indicating that they had earned an advanced degree. PACER, Progressive Aerobic Cardiovascular Endurance Run; ADHD, raw scores on the Attention Deficit Hyperactivity Disorder Rating Scale IV.*

### Fitness Assessment

The PACER was performed following the procedure described by Leger et al. (1988). During this test, participants were instructed to run back and forth between two lines 20 m apart, paced by a tone on a CD player signaling when they need to reach the opposite line. The initial speed was set at 8.5 km/h, with the speed increasing by 0.5 km/h every minute, and the test ended when participants failed to reach the end lines in the time allotted on two consecutive occasions. The total number of laps was recorded. To exclude age- and sex-related differences, we calculated age- and sex-specific percentile scores as an index of aerobic fitness based on normative data provided by the Japanese Ministry of Education, Culture, Sports, Science, and Technology (2012).

### Laboratory Procedure

After informed consent was obtained, participants’ height and weight were measured using a Tanita WB-3000 digital scale (Tanita Corp., Tokyo, Japan). Participants’ legal guardians completed the Attention Deficit Hyperactivity Disorder Rating Scale IV (DuPaul et al., 1998) and the Physical Activity Readiness Questionnaire (Thomas et al., 1992) to screen for any previous health issues that might be exacerbated by exercise. Maternal educational attainment was assessed as a proxy for socioeconomic status (Noble et al., 2007; Stevens et al., 2009), given that socioeconomic status has been associated with both cognitive control (Mezzacappa, 2004) and fitness (Freitas et al., 2007). Participants were then fitted with a 64-channel headcap with Ag/AgCl active electrodes (BioSemi ActiveTwo system, Amsterdam, the Netherlands) and were seated in a sound-attenuated room where the AX-CPT was administered. Participants were then given instructions and engaged in practice trials prior to the start of testing. The PACER was performed on a different day.

### AX-CPT

Stimuli for the present child-friendly version of the AX-CPT were clip-art images of animals (monkey and cat) that served as the cue and fruits (apple and strawberry) that served as the probe. The monkey, cat, apple, and strawberry images corresponded to target cue “A,” non-target cue “B,” target probe “X,” and non-target probe “Y,” respectively. This task required participants to press a button using their right index finger for target trials (i.e., AX trials), which occurred when the target probe was preceded by the target cue. Non-target trials, which required a button press with the right middle finger, consisted of three types: AY trials, in which the non-target probe was preceded by the target cue; BX trials, in which the target probe was preceded by the non-target cue; and BY trials, in which the non-target probe was preceded by the non-target cue. It has been documented that CNV is a composite of a readiness potential (*Bereitschaftspotential*) and stimulus-preceding negativity, which are considered to reflect motor preparation and anticipatory attention, respectively (van Boxtel and Brunia, 1994; Brunia and van Boxtel, 2001). The readiness potential has a contralateral preponderance (Shibasaki and Hallett, 2006) and can be affected by movement side and extremity (Damen et al., 1996). To exclude effects of movement-related differences in cortical activities, we asked participants to press the button using the same finger for AY and BX trials. The stimulus-response mapping (i.e., index and middle fingers for target and non-target, respectively) was the same as that used in prior studies of proactive vs. reactive mode of cognitive control during the AX-CPT (Paxton et al., 2008; Strang and Pollak, 2014). The majority of trials were AX trials (64%), and non-target trials were equiprobable (12%). After 40 practice trials, participants completed 400 trials (100 trials × 4 blocks). The viewing distance was 1 m and the stimuli subtended horizontal and vertical visual angles of 4.0°. The cue was presented for 200 ms and the probe was presented until a response was made or for 2000 ms, with a fixed stimulus onset asynchrony of 1700 ms (from cue onset to probe onset) and a randomized inter-trial interval between 1300 and 1700 ms (mean = 1500 ms from probe offset to cue onset). Total task duration was approximately 24 min (6 min × 4 blocks).

### ERP Recording

Electroencephalographic activity was measured from 64 electrode sites arranged in an extended montage based on the International 10–10 system (Chatrian et al., 1985), as well as two electrodes on the right and left mastoids. Additional electrodes were placed above and below the right orbit and on the outer canthus of each eye to monitor electrooculographic activity via bipolar recording. Continuous data were digitized at a sampling rate of 1024 Hz with a bandwidth of DC to 208 Hz (-3 dB/octave), using the BioSemi Active Two system. Oﬄine EEG processing, which was performed using Brain Vision Analyzer 2 software (Brain Products, Gilching, Germany), included re-referencing to average mastoids, low-pass filtering (10 Hz, 24 dB/octave), creation of cue-locked epochs (-500 to 3000 ms relative to cue onset), eye movement correction using the procedure described by Gratton et al. (1983), baseline correction (-100 to 0 ms relative to stimulus onset), and artifact rejection (epochs with signals that exceeded ± 100 μV). Trials with a response error were excluded from the ERP as well as reaction time (RT) analyses. For each ERP component, we normalized the data using the vector scaling procedure (McCarthy and Wood, 1985) and performed a topographic analysis. Across groups, means of 30 and 29 trials were averaged for the AY and BX trial types, respectively. Mean amplitudes were calculated in the following time windows: 600–1100 ms (cue-P3) and 1500–1700 ms (CNV) after cue onset^2^, and 250–400 ms (probe-N2) and 400–700 ms (probe-P3) after probe onset.

### Statistical Analysis

In keeping with prior studies of proactive vs. reactive mode of cognitive control during the AX-CPT (Paxton et al., 2006; Edwards et al., 2010), we focused our analyses on AY and BX trials. Response accuracy and RTs were analyzed using 2 (Group: lower-fit, higher-fit) × 2 (Trial: AY, BX) repeated measures ANOVAs. Based on our *a priori* hypotheses, we performed planned comparisons to examine the group differences within each trial type (i.e., unpaired *t*-tests) and the trial-type effect within each group (i.e., paired *t*-tests). Topographical analyses were performed to test whether scalp distributions of each ERP component differed between groups and/or trials. The amplitudes of each ERP component (cue-P3, CNV, probe-N2, and probe-P3) were analyzed using a 2 (Group) × 2 (Trial) × 5 (Site: Fz, FCz, Cz, CPz, Pz) repeated measures ANOVA. When significant interactions including the site factor were found, we normalized the data using a vector scaling procedure (McCarthy and Wood, 1985) and performed the same repeated measures ANOVAs to check whether the interaction remained significant after vector scaling. Based on Ruchkin et al. (1999), scaled data were only used to check the interaction. Scaling was performed at the same electrode sties as those used in the ANOVAs. Based on the topographical analyses, cue-P3, probe-N2, and probe-P3 amplitudes were, respectively, assessed at the CPz, Fz, and Pz electrode sites, where each reached its topographic maximum, using 2 (Group) × 2 (Trial) repeated measures ANOVAs. Based on our *a priori* hypothesis, CNV amplitude was assessed at the Fz and Cz electrode sites, using a 2 (Group) × 2 (Trial) × 2 (Site: Fz, Cz) ANOVA. Planned and *post hoc* comparisons were conducted using Bonferroni corrected *t*-tests. All statistical analyses were conducted using a significance level of *p* = 0.05.

## Results

### Task Performance

**FIGURE 1:**
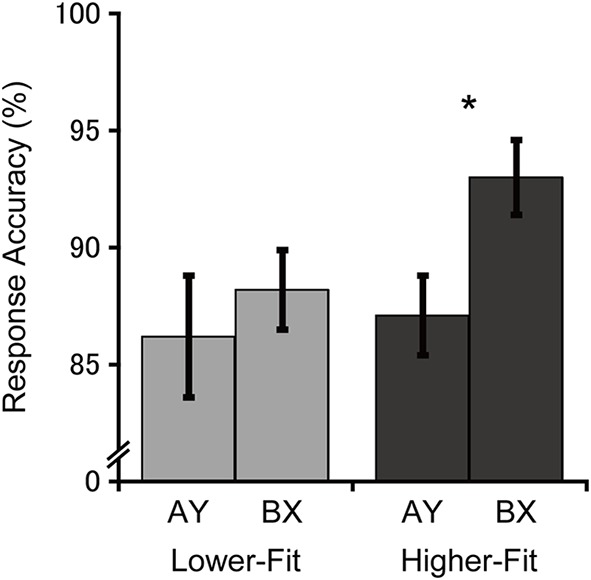
**Mean (*SE*) response accuracy for each group and trial type.** Bonferroni-corrected planned comparisons between trial types: ^∗^*p* < 0.025.

**Figure 1** illustrates response accuracies for each group and trial type. Analysis of response accuracy revealed a main effect of Trial, *F*(1, 36) = 4.6, *p* = 0.04, $\eta_{p}^{2}$ = 0.11, with greater response accuracy for the BX relative to the AY trials. No main effect or interaction involving the Group factor was observed, *F*s(1, 36) ≤ 1.9, *p*s ≥ 0.17, $\eta_{ps}^{2}$ ≤ 0.05. Bonferroni-corrected planned comparisons (*p* < 0.025) examining the trial-type effect within each group revealed that the higher-fit group had greater response accuracy for the BX relative to the AY trial type, *t*(18) = 2.6, *p* = 0.02, whereas no such difference was observed for the lower-fit group, *t*(18) = 0.7, *p* = 0.50. Bonferroni-corrected planned comparisons (*p* < 0.025) examining group differences within each trial type revealed no significant group differences for the BX trials, *t*(36) = 2.1, *p* = 0.05 (after Bonferroni correction), or the AY trials, *t*(36) = 0.3, *p* = 0.78.

Analysis of RTs revealed a main effect of Trial, *F*(1, 36) = 256.0, *p* < 0.001, $\eta_{p}^{2}$ = 0.88, with shorter RTs for the BX (mean = 347.8 ms, *SE* = 19.8) relative to the AY (mean = 600.4 ms, *SE* = 22.0) trials. No main effect or interaction involving the Group factor was observed, *F*s(1, 36) ≤ 1.1, *p*s ≥ 0.31, $\eta_{ps}^{2}$ ≤ 0.03. Planned comparisons revealed no significant group differences.

### ERPs

**FIGURE 2:**
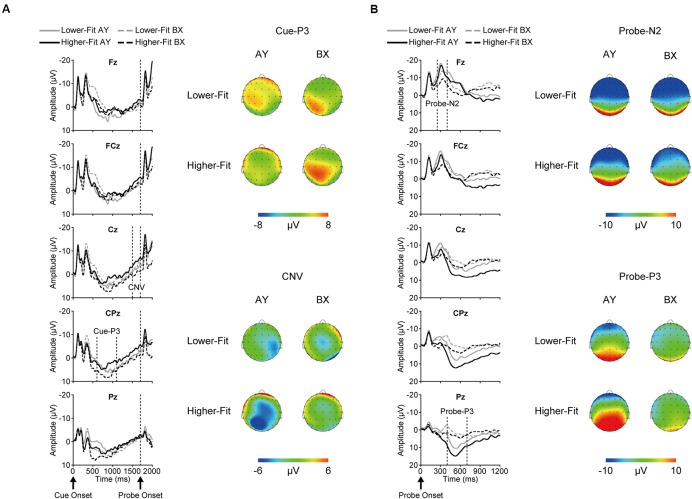
**Grand average event-related brain potential (ERP) waveforms and topographical maps for each ERP component after cue onset (A) and probe onset (B) for each group and trial type**.

**Figure 2** illustrates grand average ERP waveforms after cue onset (A) and probe onset (B) for each group and trial type.

### Cue-P3

Topographic analysis of cue-P3 revealed a main effect of Site, *F*(4, 144) = 13.0, *p* < 0.001, $\eta_{p}^{2}$ = 0.27, indicating a centro-parietal distribution (i.e., Fz, FCz < Cz, CPz, Pz). The Trial × Site interaction was significant, *F*(4, 144) = 9.0, *p* < 0.001, $\eta_{p}^{2}$ = 0.20. This interaction remained significant after scaling, *F*(4, 144) = 4.5, *p* = 0.02, $\eta_{p}^{2}$ = 0.11. *Post hoc* analysis for the BX trials revealed a main effect of Site, *F*(4, 148) = 22.1, *p* < 0.001, $\eta_{p}^{2}$ = 0.37, indicating a centro-parietal distribution (i.e., Fz, FCz < Cz, Pz < CPz), whereas no such difference was observed for the AY trials, *F*(4, 148) = 0.9, *p* = 0.39, $\eta_{p}^{2}$ = 0.03.

**FIGURE 3:**
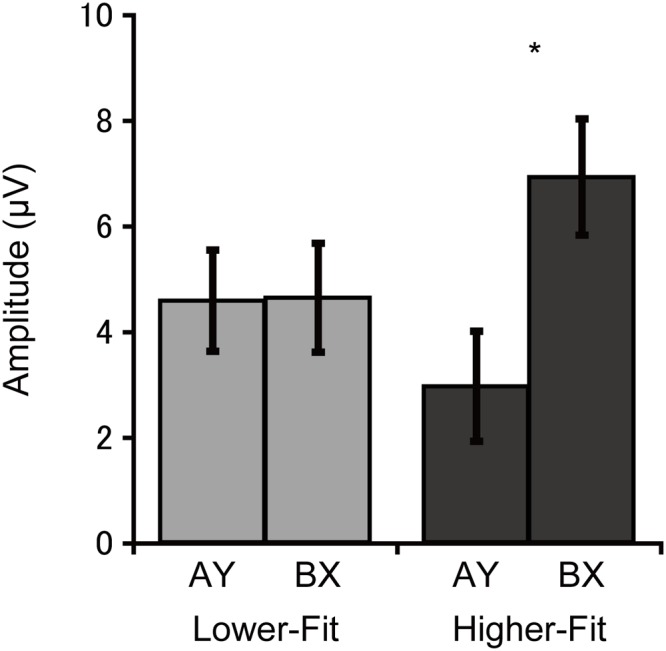
**Mean (*SE*) cue-P3 amplitude for each group and trial type collapsed across electrode sites, illustrating the Group × Trial interaction**. Bonferroni-corrected *post hoc* comparisons between trial types: ^∗^*p* < 0.025.

Cue-P3 amplitude was assessed at the CPz electrode site. Analysis of cue-P3 amplitude revealed a main effect of Trial, *F*(1, 36) = 5.3, *p* = 0.03, $\eta_{p}^{2}$ = 0.13, which was qualified by a Group × Trial interaction, *F*(1, 36) = 5.0, *p* = 0.03, $\eta_{p}^{2}$ = 0.12. Bonferroni-corrected *post hoc t*-tests (*p* < 0.025) indicated larger cue-P3 amplitudes for the BX relative to the AY trials for the higher-fit group, *t*(18) = 3.1, *p* = 0.007, while no such difference was observed for the lower-fit group, *t*(18) = 0.05, *p* = 0.96. **Figure 3** illustrates this interaction.

### CNV

**FIGURE 4:**
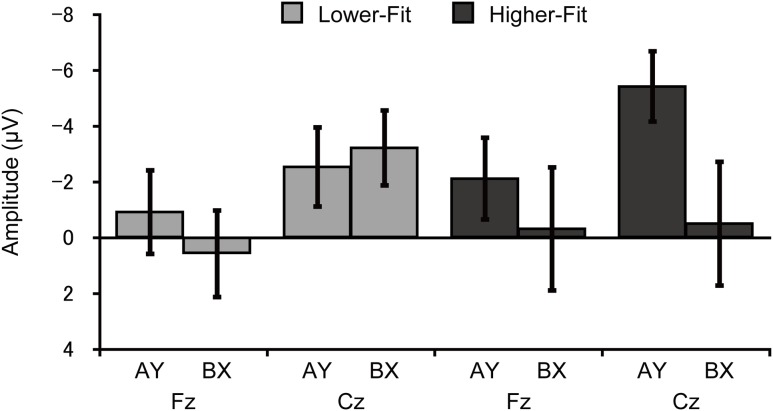
**Mean (*SE*) CNV amplitude for each trial type and site for the lower-fit and higher-fit group, illustrating the Group × Trial × Site interaction**.

Topographic analysis of CNV revealed a main effect of Site, *F*(4, 144) = 3.6, *p* = 0.02, $\eta_{p}^{2}$ = 0.09, indicating smaller amplitude at Fz relative to FCz. No other main effects or interactions were observed, *p*s ≥ 0.12. CNV amplitude was assessed at the Fz and Cz electrode sites based on our *a priori* hypothesis. Analysis of CNV amplitude revealed a main effect of Site, *F*(1, 36) = 8.4, *p* = 0.006, $\eta_{p}^{2}$ = 0.19, which was qualified by a Group × Trial × Site interaction, *F*(1, 36) = 5.2, *p* = 0.03, $\eta_{p}^{2}$ = 0.13. **Figure 4** illustrates this 3-way interaction. As can be seen in **Figure 4**, for the higher-fit group, CNV amplitude was larger for the AY relative to the BX trials at Cz, although this difference failed to reach statistical significance, *t*(18) = 2.0, *p* = 0.07. No such difference was observed at Fz, *t*(18) = 0.8, *p* = 0.42, or for the lower-fit group, *t*s(18) ≤ 1.3, *p*s ≥ 0.21.

We also performed correlation analyses between CNV amplitude at the Cz electrode site and task performance measures (i.e., response accuracy and RT) to support interpretation of the differences in CNV amplitude between groups. Results indicated that larger CNV amplitude was related to greater response accuracy for AY trials, *r* = -0.33, *p* = 0.04, whereas no such relationship was observed for BX trials, *r* = -0.13, *p* = 0.43. By contrast, CNV amplitude was not related to RT for AY trials, *r* = 0.27, *p* = 0.11, whereas smaller CNV amplitude was related to shorter RT for BX trials, *r* = 0.47, *p* = 0.003.

### Probe-N2

Topographic analysis of probe-N2 revealed a main effect of Site, *F*(4, 144) = 92.4, *p* < 0.001, $\eta_{p}^{2}$ = 0.72, with a topographic maximum at Fz (i.e., Fz > FCz > Cz > CPz > Pz). Although the Trial × Site interaction was significant, *F*(4, 144) = 19.2, *p* < 0.001, $\eta_{p}^{2}$ = 0.35, this interaction was not significant after scaling, *F*(4, 144) = 1.8, *p* = 0.19, $\eta_{p}^{2}$ = 0.05.

Probe-N2 amplitude was assessed at the Fz electrode site. Analysis of probe-N2 amplitude revealed a main effect of Trial, *F*(1, 36) = 11.2, *p* = 0.002, $\eta_{p}^{2}$ = 0.24, with larger probe-N2 amplitude for AY relative to BX trials. The main effect and interaction involving the Group factor were not significant, *F*s(1, 36) ≤ 1.2, *p*s ≥ 0.29, $\eta_{ps}^{2}$ ≤ 0.03.

### Probe-P3

Topographic analysis of probe-P3 revealed a main effect of Site, *F*(4, 144) = 47.7, *p* < 0.001, $\eta_{p}^{2}$ = 0.57, indicating a centro-parietal distribution (i.e., Fz < FCz < Cz < Pz; Fz < FCz < Cz < CPz). Although the Trial × Site interaction was significant, *F*(4, 144) = 58.2, *p* < 0.001, $\eta_{p}^{2}$ = 0.62, this interaction was not significant after scaling, *F*(4, 144) = 0.5, *p* = 0.58, $\eta_{p}^{2}$ = 0.01.

Probe-P3 amplitude was assessed at the Pz electrode site. Analysis of probe-P3 amplitude revealed a main effect of Trial, *F*(1, 36) = 61.9, *p* < 0.001, $\eta_{p}^{2}$ = 0.63, with larger probe-P3 amplitude for AY relative to BX trials. The main effect and interaction involving the Group factor were not significant, *F*s(1, 36) ≤ 2.5, *p*s ≥ 0.13, $\eta_{ps}^{2}$ ≤ 0.06.

## Discussion

As hypothesized, higher-fit children exhibited greater response accuracy for the BX trials (non-target cue – target probe) relative to the AY trials (target cue – non-target probe), whereas lower-fit children had comparable response accuracies for the two trial types. In terms of the dual mechanisms of control framework, proactive control, which is cue-driven, is beneficial for BX performance but results in deteriorated AY performance (Paxton et al., 2006; Edwards et al., 2010; Braver, 2012). Accordingly, the current behavioral findings support the hypothesis that higher-fit children can engage in more effective proactive control relative to lower-fit children, in order to adapt to the nature of the cognitive task at hand.

Neuroelectric data support the behavioral findings. Higher-fit children had larger cue-P3 amplitudes for the BX relative to AY trials, whereas lower-fit children exhibited comparable cue-P3 amplitudes for the two trial types. These findings suggest that higher-fit children recruited a greater amount of attentional resources to the B (non-target) cue, probably because they made response choices upon the B cue presentation. For the AY trials, response choices could not be made upon the A (target) cue presentation, and thus higher-fit children may have allocated a lesser amount of attentional resources to the A cue. By contrast, lower-fit children may not have been able to utilize cue information effectively; therefore cue-P3 amplitude did not differ between the trial types.

Contrary to our hypothesis, higher-fit children had larger CNV amplitude for the AY relative to BX trials, and this difference was selectively observed at the central electrode site. As mentioned in the Introduction, several studies have suggested that frontal CNV is associated with cognitive preparation processes, whereas central CNV is associated with response preparation processes (Leynes et al., 1998; Lorist et al., 2000; Falkenstein et al., 2003; Wild-Wall et al., 2007; Kamijo et al., 2010, 2011). It is therefore plausible that the larger CNV for the AY trials reflects more effective response preparation, rather than cognitive preparation, for the higher-fit children. These findings suggest that higher-fit children may have activated response preparation processes for the AY probes, since response choice was required upon presentation of the probe. For the BX trials, they may have already made a decision about their response (i.e., a button press with the middle finger) before the CNV time window, as reflected by larger cue P3 amplitude (i.e., greater utilization of cue information), allowing for more efficient response preparation, as reflected in smaller CNV. By contrast, lower-fit children again exhibited comparable CNV amplitude between the trial types, suggesting that they may have exerted less effort in response preparation, partly due to their lower ability to utilize cue information.

Taken together, these ERP findings suggest that greater childhood fitness is associated with more effective utilization of cue information and response preparation more appropriate to trial type. This in turn may have resulted in superior task performance on the BX trials. It is noteworthy that higher-fit children did not exhibit worse AY performance relative to lower-fit children, irrespective of their utilization of proactive control. We believe that this is also because greater fitness is associated with an enhanced ability to flexibly modulate contextual processing and more effective response preparation. This interpretation is supported by the results of correlation analyses. For the AY trials, larger CNV was related to greater response accuracy, suggesting that enhanced response preparation resulted in superior task performance. By contrast, for the BX trials, smaller CNV was related to shorter RT, suggesting that more efficient response preparation led to superior task performance.

Note that several adult studies have demonstrated that higher-fit individuals exhibit smaller CNV amplitudes relative to their lower-fit peers, suggesting that greater aerobic fitness is associated with more efficient task preparation (Hillman et al., 2002; Kamijo et al., 2010). These results are inconsistent with our hypothesis that greater fitness is associated with larger CNV, but partially consistent with the present data. Hillman et al. (2002) used an S1-S2-S3 task, in which participants were required to make a decision about their response (i.e., left or right) upon the S2 presentation and to press one of two buttons upon the S3 presentation (i.e., the imperative stimulus). The nature of this cognitive task is similar to the BX trials of the present study. That is, when participants are able to make a decision about their response before presentation of an imperative stimulus, more efficient response preparation, which results in superior task performance, would be reflected in smaller CNV. By contrast, when the decision making is required upon presentation of an imperative stimulus, as in the AY trials in the present study, enhanced response preparation, as denoted by larger CNV, may lead to superior task performance. However, Kamijo et al. (2010) showed that higher-fit young adults had smaller CNV even though the decision making was required upon presentation of an imperative stimulus in that task. They also indicated that the relationship between fitness and CNV amplitude was observed during speed instructions (to respond as quickly as possible), but not during accuracy instructions (to respond as accurately as possible). Given that both speed and accuracy were stressed for AX-CPT performance in the present study, these differences in task instructions may be related to the discrepancy. In addition, given that developmental studies have indicated that CNV amplitude increases with age (Bender et al., 2002; Segalowitz and Davies, 2004), the difference in participants’ age (children vs. young adults) also should influence the relationship between fitness and CNV. Given this evidence, it is likely that the direction of differences in CNV amplitude based on fitness levels differs based on the nature of the cognitive tasks, the task instructions, and the participants’ age.

Lastly, probe-elicited N2 and P3 did not differ between groups. That is, the greater response accuracy for BX relative to AY trials observed in the higher-fit group was not due to cognitive processing after probe presentation; as discussed above, this difference was attributable to cognitive processing and response preparation processing before probe presentation. As explained in the Introduction, proactive control is characterized by future-oriented early selection that anticipates and prevents interference before it occurs (Braver et al., 2007; Braver, 2012). Thus, the results of probe-elicited ERP components support our contention that greater childhood fitness is associated with more effective utilization of proactive control.

### Limitations

It should be noted that because we employed a cross-sectional design, the present findings do not support a causal relationship between childhood fitness and cognitive control mode. Although a longitudinal, randomized controlled intervention study (Kamijo et al., 2011) suggested that aerobic fitness training results in a mode shift from reactive to proactive control, that study did not manipulate cognitive control mode. Further longitudinal studies are needed to shed light on how increases in childhood fitness influence reliance on proactive vs. reactive modes of cognitive control. Another limitation of the present study is that the median-split procedure used to bifurcate participants into lower-fit and higher-fit groups can obscure differences in cognitive control abilities based on participants’ fitness levels (Hillman et al., 2012). The present findings indicate differences in response accuracy and ERP component amplitudes between trial types within each group, but not group differences on those measures within each trial type. The lack of group differences in the present study may be attributable to use of the median-split procedure. Finally, we did not employ measures of cognitive development such as IQ. However, given that none of the participants received special education services related to cognitive or attentional disorders, and overall response accuracy on the AX-CPT was quite high (>85%), it is reasonable to conclude that children in the present study had normal cognitive development. Thus, it is unlikely that children’s cognitive developmental status affected the current results. Nonetheless, future studies should include measures of cognitive development.

## Conclusion

The present behavioral performance measures confirm the hypothesis that greater childhood fitness is associated with more effective utilization of proactive control. The ERP findings support this notion, and further suggest that greater fitness is associated with better ability to flexibly modulate context processing, and more specifically, with more effective utilization of cue information and response preparation more appropriate to trial type. The present study provides novel insights into the relationship between fitness and cognition from the perspective of cognitive control mode during task preparation.

## Author Contributions

KK contributed to the design of the study, the acquisition of data, conducted the statistical analyses, interpreted the data for the study, and drafted the manuscript. HM interpreted the data for the study, and critically revised the manuscript. All authors approved the final version of the manuscript.

## Conflict of Interest Statement

The authors declare that the research was conducted in the absence of any commercial or financial relationships that could be construed as a potential conflict of interest.

## Footnotes

1 Although all trials required a response in the present study, AY, BX, and BY trials are referred to as non-target trials, in keeping with prior studies (Paxton et al., 2006; Edwards et al., 2010).

2 We also assessed cue-P2, but this component amplitude did not differ between groups. Thus discussion of cue-P2 is not included in the present paper. However, because cue-P2 amplitude differed between AY and BX trials, these results are provided in the Supplementary Figure S1.
