# Supplementary material for: Fitness and ERP Indices of Cognitive Control Mode during Task Preparation in Preadolescent Children
Source: Front Hum Neurosci. 2016 Aug 30;10:441. doi: 10.3389/fnhum.2016.00441 (PMC5003924; doi:10.3389/fnhum.2016.00441)
Supplement: Supplementary file 1 [file Image_1.PDF]

Supplementary Material

Fitness and ERP indices of cognitive control mode during task preparation in preadolescent children

Keita Kamijo*, Hiroaki Masaki

*** Correspondence:** Keita Kamijo: k-kamijo@aoni.waseda.jp

1 Supplementary Figure

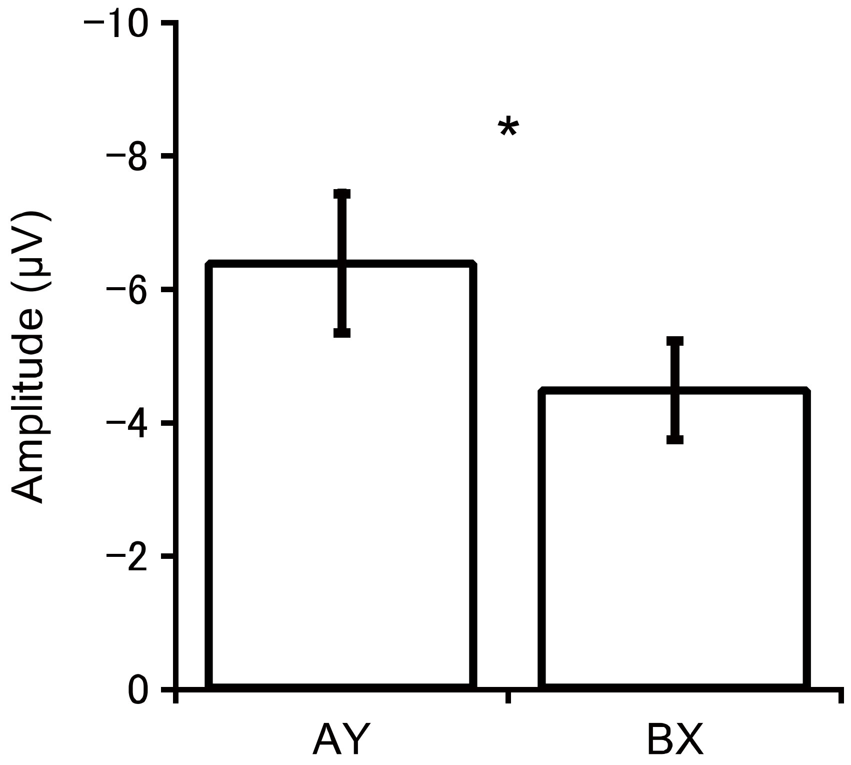

Supplementary Figure 1. Mean (*SE*) cue-P2 amplitude for each trial type collapsed across groups. Cue-P2 was evaluated as the mean amplitude between 150 and 300 ms after cue onset. Cue-P2 amplitude was assessed at the Fz electrode site, using a 2 (Group) × 2 (Trial) repeated measures MANOVA. Analysis of cue-P2 amplitude revealed a main effect of Trial, $F(1, 36) = 6.3$, $p = .02$, $\eta^2p = .15$, with larger (or less negative) cue-P2 amplitude for BX relative to AY trials. No main effect or interaction involving the Group factor was observed, $Fs(1, 36) < 0.5$, $ps > .47$, $\eta^2ps < .02$.
